# Explorative analysis of a score predicting the therapy response of patients with metastatic, castration resistant prostate cancer undergoing radioligand therapy with ^177^Lu-labeled prostate-specific membrane antigen

**DOI:** 10.1007/s12149-020-01567-3

**Published:** 2020-12-22

**Authors:** Kai Huang, Imke Schatka, Julian M. M. Rogasch, Randall L. Lindquist, Maria De Santis, Barbara Erber, Piotr Radojewski, Winfried Brenner, Holger Amthauer

**Affiliations:** 1 Department of Nuclear Medicine, Charité-Universitätsmedizin Berlin, Corporate member of Freie Universität Berlin, Humboldt-Universität Zu Berlin and Berlin Institute of Health, Augustenburger Platz 1, 13353 Berlin, Germany; 2 Department of Urology, Charité-Universitätsmedizin Berlin, Corporate member of Freie Universität Berlin, Humboldt-Universität Zu Berlin and Berlin Institute of Health, Charitéplatz 1, 10117 Berlin, Germany

**Keywords:** Predictive score, Radioligand therapy, Metastatic castration-resistant prostate cancer, ^177^Lu PSMA

## Abstract

**Objective:**

Up to 60% of patients with metastatic, castration-resistant prostate cancer (mCRPC) treated with ^177^Lu prostate-specific membrane antigen (PSMA) radioligand therapy (RLT) achieves a partial biochemical response with a decrease of > 50% in prostate-specific antigen (PSA) levels. The remaining fractions, however, do not respond to RLT. The aim of this explorative analysis was to identify pre-therapeutic factors for the prediction of response.

**Methods:**

46 patients [age = 68 years (50–87)] with mCRPC who consecutively underwent RLT with ^177^Lu PSMA [median applied activity = 6 GBq (2.9–6.2)] were included and analysed retrospectively. The association of different clinical and laboratory factors and parameters from pre-therapeutic ^68^Ga PSMA positron emission tomography (PET) with the outcome of RLT was tested (Fisher’s test). Outcome was defined as PSA changes 8 weeks after second RLT [partial response (PR), PSA decrease > 50%; progressive disease (PD), PSA increase ≥ 25%; stable disease (SD), others]. Significant predictive factors were combined in a predictive score.

**Results:**

30% showed a post-treatment PR (median 73% PSA decrease), 35% SD (median 17% PSA decrease) and 35% PD (median 42% PSA increase). Significant predictors for PD were alkaline phosphatase (ALP) > 135 U/l (*p* = 0.002), PSA > 200 ng/ml (*p* = 0.036), and maximum standardized uptake value (SUVmax) of the “hottest lesion” in pre-therapeutic PET < 45 (*p* = 0.005). The predictive score including PSA, ALP and SUVmax could separate 2 distinct groups of patients: ≤ 2 predictive factors (19% PD) and 3 predictive factors (90% PD).

**Conclusion:**

The presented predictive score allowed a pre-therapeutic estimate of the expected response to 2 cycles of RLT. As our study was retrospective, prospective trials are needed for validation.

## Introduction

Prostate cancer has the highest incidence and the third highest cancer related mortality for men in Europe [1]. In the last decade, several treatment options for patients with metastatic, castration-resistant prostate cancer (mCRPC) have become established, including abiraterone, enzalutamide, docetaxel and cabazitaxel [2]. More recently, radioligand therapy (RLT) with ^177^Lu prostate-specific membrane antigen (PSMA) was developed and has shown promising results with regard to prostate-specific antigen (PSA) response rates and radiographic responses [3]. Retrospective as well as early prospective data confirmed the favourable safety profile of RLT [4, 5].

Although up to 60% of the patients showed some degree of response to RLT, some do not benefit from this treatment [4, 5]. The presence of visceral metastases, alkaline phosphatase (ALP) ≥ 220 U/L [5], high platelet count, a regular need for analgesics [6] or elevated lactate dehydrogenase [7] has been identified as a negative predictor for the outcome, whereas ALP < 220 U/L, a cumulative injected activity ≥ 18.8 GBq [8], albumin ≥ 38.6 g/L, aspartate transaminase (AST) ≤ 24 U/L, haemoglobin ≥ 10.4 g/dL, absence of liver metastases [9], and higher standardized uptake value (SUV) mean or max in positron emission tomography (PET) with PSMA ligands have been reported to be associated with favourable outcomes [10]. However, the predictive value of these factors varies significantly between studies.

The aim of this study was first, to identify predictive factors for the outcome of RLT with ^177^Lu PSMA considering laboratory parameters, medical history and imaging obtained before the first RLT and second, to derive a predictive score which can simplify the selection of patients who are or are not likely to benefit from RLT.

## Materials and methods

This single-institution retrospective study was approved by the institutional ethics review board (EA2/177/17). All patients signed a written informed consent form for the study.

### Patients

Between 06/2015 and 12/2018, 91 patients with mCRPC consecutively underwent RLT with ^177^Lu PSMA in the department of nuclear medicine of our university hospital.

Inclusion criteria were: (1) at least two cycles of RLT with ^177^Lu PSMA, (2) ^68^Ga PSMA positron emission tomography (PET)/computed tomography (CT) examination in the same department ≤ 8 weeks prior to first cycle of RLT and (3) complete patient records including follow-up for at least 2 months after second cycle RLT. 45 patients were excluded for the following reasons: PSMA-PET prior to RLT in other facilities or non-PET imaging with PSMA ligands (*n *= 43) and absence of follow-up after RLT (*n* = 2), Thus, the final study population included 46 patients.

Patients records were assessed for the medical history including age at the time point of RLT, time between first diagnosis and RLT, initial Gleason score, initial D’Amico risk classification [11], presence of distant metastases at the time of initial diagnosis, treatment before RLT and type of metastases at time of RLT. Baseline laboratory tests including liver, renal and hematologic function, electrolytes, PSA and testosterone levels were recorded. Imaging parameters from ^68^Ga PSMA PET/CT including maximum and mean standard uptake value (SUVmax and SUVmean) of the lesion with the highest tracer uptake and the visual tumor burden were also determined. Visual tumor burden was subdivided into three categories: low (number of focal lesions in PET/CT < 10), medium (number of lesions in PET/CT > 10 and < 30) and high (disseminated metastatic spread).

### Treatment, imaging, laboratory and response assessment

RLT with ^177^Lu PSMA and imaging with ^68^Ga PSMA PET/CT were performed according to consensus recommendations or as previously described [12, 13]. Laboratory parameters for liver, renal and hematologic function, electrolytes, PSA and testosterone levels were collected at each RLT cycle. In line with the German multicenter study for RLT with ^177^Lu PSMA [5], evaluation of the treatment response was based on the change in PSA levels between baseline at the first RLT cycle and two months after the second RLT cycle. Treatment response was defined according to Prostate Cancer Clinical Trials Working Group 2 and 3 as follows: partial response (PR) > 50% decline; progressive disease (PD) ≥ 25% increase; stable disease (SD) ≤ 50% decline and < 25% increase of PSA level [14, 15].

### Statistical analysis

Statistical analyses were performed using SPSS 25 (IBM Corporation, Armonk, NY, USA). To evaluate the association between single factors and PD under RLT, receiver operating characteristic (ROC) curve analysis was performed for each factor. For factors with an area under the curve (AUC) > 0.65 or < 0.35 and a *p* value < 0.05, the optimal cut-off value was determined (Youden’s index) for binarization, and Fisher’s exact test for PD vs. SD/PR was performed. P values for each factor were corrected for multiple comparisons using the Benjamini–Hochberg procedure [16]. A *p* value < 0.05 was considered significant. The combined predictive score included all single factors with equal weighting that had shown significant association with PD in Fisher’s exact test. Additionally, all factors that were included in the predictive score, were included in a binary logistic regression analysis (PD vs. SD/PR) to examine their independent association with PD.

## Results

### Patient, treatment and response data

The median patient age was 68 years (range 50–87). The median PSA value before RLT was 79 ng/ml (range 4–3133). Patient characteristics including previous treatments and relevant baseline parameters are summarized in Table 1.

**Table 1:** Patient characteristics

Characteristic	Number (% or range)
Total patient number	46
Median age (years)	68 (50–87)
Median baseline prostate-specific antigen (PSA) (ng/mL)	79 (4–3133)
Median Gleason score	9 (6–10)
Initial D’Amico group	
Low-risk	3 (6)
Intermediate-risk	4 (9)
High-risk	39 (85)
Median time since first diagnosis (months)	60 months (10–140)
Extent at first diagnosis	
Localized	30 (65)
Distant metastasized	16 (35)
Previous therapy	
Radical prostatectomy	24 (52)
Radiation therapy	26 (57)
Androgen-deprivation therapy	46 (100)
Abiraterone	28 (61)
Enzalutamide	26 (57)
Chemotherapy	29 (63)
Radium-223 or/and radiotherapy (bone)	14 (30)
Others (including e.g. immunotherapy)	8 (17)
Site of metastases at first radioligand therapy	
Lymph nodes	39 (85)
Bone	44 (96)
Liver	7 (15)
Visceral including liver	14 (30)
Median baseline alkaline phosphatase (U/L)	119 (54–1188)
Median baseline maximum standardized uptake value (SUVmax)	47 (5–133)
Median baseline mean standardized uptake value (SUVmean)	21 (4–49)
Visual tumor burden	
Low	18 (39)
Median	10 (22)
High	18 (39)

Overall, 117 treatment cycles were performed with a median activity of 6 GBq (range 2.9–6.2) ^177^Lu PSMA per cycle. 3 patients underwent four, 19 patients three and 24 patients underwent two RLT cycles.

After the second RLT cycle, fourteen patients (30%) demonstrated PR with median PSA decrease of 73%, sixteen patients (35%) showed SD with median PSA decrease of 17%, and sixteen patients (35%) developed PD with median PSA increase of 42%. The median overall survival was 19.4 months (range 5.0–42.5) from the first RLT cycle.

### Predictors of treatment response

Among all the analysed parameters, only PSA, ALP, SUVmax, SUVmean, therapy activity per RLT cycle and cumulative therapy activity fulfilled the predefined selection criteria as possible negative predictors. Results of the ROC analysis are summarized in Table 2. Although lower therapy activity per RLT cycle and lower cumulative therapy activity are significantly associated with PD in ROC curve analysis, neither parameter is included in the predictive score, since these parameters are unknown before the first RLT cycle and could therefore not be included as predictive factors into treatment decisions.

**Table 2:** Receiver operating characteristic **(**ROC) curve analysis for progressive disease after radioligand therapy

Parameter	Area under the curve (AUC; 95% confidence interval)	*p* value
Age	0.59 (0.43–0.76)	0.299
Time since first diagnosis	0.58 (0.41–0.75)	0.393
Gleason score at first diagnosis	0.41 (0.23–0.59)	0.299
D’Amico risk at first diagnosis	0.42 (0.24–0.60)	0.393
Extent at first diagnosis	0.47 (0.30–0.65)	0.764
Abiraterone	0.66 (0.49–0.82)	0.080
Enzalutamide	0.52 (0.34–0.69)	0.854
Chemotherapy	0.58 (0.41–0.76)	0.356
**Therapy activity per RLT cycle**	**0.32 (0.14–0.51)**	**0.049**
**Cumulative therapy activity**	**0.25 (0.10–0.40)**	**0.006**
Lymph node metastases	0.43 (0.25–0.61)	0.406
Bone metastases	0.53 (0.36–0.41)	0.712
Liver metastases	0.62 (0.44–0.80)	0.174
Visceral metastases (including liver)	0.60 (0.43–0.78)	0.258
**Prostate-specific antigen (PSA)**	**0.84 (0.69–0.99)**	**0.003**
Serum creatinine	0.56 (0.39–0.73)	0.518
Aspartate transaminase	0.63 (0.45–0.80)	0.159
Alanine transaminase	0.53 (0.33–0.73)	0.747
**Alkaline phosphatase (ALP)**	**0.73 (0.54–0.93)**	**0.041**
Gamma-glutamyltransferase	0.67 (0.46–0.89)	0.130
Blood urea nitrogen	0.45 (0.28–0.63)	0.612
Uric acid	0.57 (0.29–0.85)	0.611
Leucocytes	0.42 (0.24–0.59)	0.344
Erythrocytes	0.41 (0.23–0.59)	0.310
Thrombocytes	0.37 (0.20–0.54)	0.153
Hemoglobin	0.42 (0.24–0.59)	0.356
**Maximal standardized uptake value (SUVmax)**	**0.29 (0.13–0.46)**	**0.022**
**Mean standardized uptake value (SUVmean)**	**0.30 (0.14–0.47)**	**0.029**
Visual tumor burden	0.47 (0.29–0.65)	0.747

Statistically significant results are printed in bold

Table 3 shows the results of the Fisher’s exact test for PSA, ALP, SUVmax and SUVmean with their corresponding optimal cut-off values, sensitivity and specificity. All factors remained statistically significant after Benjamini–Hochberg adjustment. SUVmean was not considered in the predictive score because it did not show superior predictive value compared to the SUVmax while demonstrating lower inter-rater reliability due to the non-standardized measurement. Thus, the predictive score included baseline PSA > 200 ng/ml, ALP > 135 U/l, and SUVmax < 45, equally weighted.

**Table 3:** Fisher’s exact test for association of single variables with progressive disease (PD) after radioligand therapy (RLT)

Parameter	Patients (SD/PR)	Patients (PD)	2-sided *p* value	Sensitivity (95% CI)	Specificity (95% CI)
Prostate-specific antigen (PSA)					
> 200 ng/mL	5 (11%)	8 (18%)	0.036	50% (25–75%)	83% (65–94%)
≤ 200 ng/mL	25 (54%)	8 (17%)			
Alkaline Phosphatase (ALP)					
> 135 ng/mL	4 (9%)	10 (22%)	0.002	63% (35–85%)	87% (69–96%)
≤ 135 ng/mL	26 (56%)	6 (13%)			
Maximum standardized uptake value (SUVmax)					
< 45	10 (22%)	13 (28%)	0.005	81% (54–96%)	67% (47–83%)
≥ 45	20 (43%)	3 (7%)			
Mean standardized uptake value (SUVmean)					
< 13	4 (9%)	8 (18%)	0.013	50% (25–75%)	87% (69–96%)
≥ 13	26 (56%)	8 (17%)			

Sensitivity and specificity with their 95% confidence intervals (95% CI) are provided regarding PD vs. stable disease (SD)/partial remission (PR)

Among 46 patients, 36 showed ≤ 2 predictive factors and 10 showed 3 predictive factors. Among the 36 patients with ≤ 2 predictive factors, 29 patients (81%) had SD or PR compared to 7 patients (19%) with PD. In 10 patients with 3 predictive factors, 1 patient (10%) showed SD or PR while 9 patients (90%) developed PD (Fisher’s exact test, *p* < 0.001).

In binary logistic regression including PSA > 200 ng/ml, ALP > 135 U/l and SUVmax < 45, only SUVmax < 45 was significantly associated with PD vs. SD/PR [odds ratio (OR) 5.66; 95% confidence interval (CI) 1.03–31.0; *p* = 0.046), while PSA > 200 ng/ml (OR 3.3; 95% CI 0.6–18.4; *p* = 0.17) and ALP > 135 U/l (OR 3.9; 95% CI 0 7–20.7; *p* = 0.11] were both not significant.

## Discussion

To the best of our knowledge, although there have been several previous reports on prognostic or predictive factors for RLT with ^177^Lu PSMA in patients with mCRPC [5–10], this is the first simple clinical score to predict the outcome after the first two cycles of ^177^Lu PSMA. The score combines laboratory (PSA, ALP) and imaging (SUVmax) parameters and can identify both patients likely to benefit from RLT and potential non-responders to RLT.

MCRPC patients with higher PSA levels have a higher likelihood to develop PD under treatment than patients with lower PSA levels. Especially, a high baseline serum PSA level of > 200 ng/ml seems to be a significant predictor of an unfavorable outcome. This is consistent with the results of former studies showing that high serum PSA levels correlate strongly with the risk of prostate cancer progression, not only in the initial stage of disease, but also in advanced disease [17].

In the present study, a high ALP of > 135 U/l was also defined as a significant predictive factor for unfavorable outcome after RLT. This is in line with previous studies showing a poor outcome after RLT with ^177^Lu PSMA in patients with elevated ALP [5, 8, 18]. A similar outcome has been also reported for patients treated with chemotherapy [19]. ALP is a marker for bone turnover [19], which has led to the hypothesis that a high ALP correlates with progressive bone metastases. However, the presence of bone metastases was not a potential predictive parameter in the current analysis because almost every patient (44/46 patients) showed bone metastases, regardless of outcome. The reason why visual extent of disease did not correlate with outcome, is probably that the extent of disease could also be affected by lymph node metastases (present in 39/46 patients), which do not result in an increased ALP.

Low baseline SUVmax (< 45) in ^68^Ga PSMA PET also suggested an unfavorable outcome after RLT in the current study, which persisted in multivariable binary logistic regression after adjustment for PSA and ALP. The SUVmax/mean denotes the maximum/mean uptake in a specified region of interest (e.g., a lesion) and is the most commonly used semi-quantitative parameter in PET. This may be explained by the requirement for a targeted therapy of a minimum level of PSMA expression on the cell surface to achieve adequate lutetium internalization into the cell. The SUVmax of a radiotracer has a significant role in the diagnosis and treatment selection for different types of tumors, e.g. lymphoma [20], head and neck squamous cell carcinoma [21], pancreatic tumor [22], neuroendocrine tumors [23] or even prostate cancer [24]. A recent pilot study on 14 prostate cancer patients showed that patients with a PSA response ≥ 50% after treatment with ^177^Lu PSMA had a significantly higher baseline SUVmax in a pre-therapeutic scan with ^68^Ga PSMA-PET (44 vs. 17) [10]. This study also reported significantly higher SUVmean in patients with PSA response ≥ 50% (10 vs. 6) [10]. Likewise, SUVmean in ^68^Ga PSMA-PET was a significant predictive factor of response in the present analysis. However, it was not included in the combined predictive score because of its high inter-rater variability and limited reproducibility [25, 26]. The current study used the SUVmax of a single “hottest” lesion as a predictor of PSA response. SUVmax can be obtained intuitively, conveniently and with presumably high interobserver agreement up to 100% [25], because minimal observer interaction is required to obtain the highest SUVmax among potentially dozens of (confluent) lesions without necessitating a specific volume of interest. However, this method does not account for potential inter-lesional heterogeneity of PSMA expression or ^68^Ga PSMA uptake [27], respectively. One may note that in the most relevant patient group with low SUVmax (i.e., higher risk for PD), a low SUVmax in the “hottest” lesion is per se representative of all lesions because any other lesion in the same patient has—by definition—an even lower uptake. Furthermore, inter-lesional SUV heterogeneity in these patients is limited because “adequate uptake of PSMA ligands on the basis of a pre-therapy imaging study” in every lesion is demanded for eligibility of these patients for RLT according to current guidelines [28].

The practice of limiting assessment to a single lesion is in line with the PET Response Criteria in Solid Tumors [29] criteria, where only the “hottest single tumor lesion” is defined as the “target lesion”.

Not all potential imaging parameters were examined in our study, e.g. textural heterogeneity was recently postulated as a predictive factor in ^177^Lu PSMA treatment [30]. Our goal was however, to derive a predictive score that is simple and intuitive for clinical use. Hence, only parameters which are determined routinely and quickly before treatment were analyzed and included.

Clear limitations of this study are the retrospective design and small sample size, which may explain why predictive factors reported by other studies [5–10], e.g. blood count parameters or visceral metastases, were not significant in our analyses. Moreover, multivariable binary logistic regression did not prove the independent predictive value of PSA and ALP in addition to SUVmax—despite previous studies demonstrating their importance in the context of ^177^Lu PSMA treatment [5, 8, 9]. The observed odds ratios of approximately 3 to 4 and *p* values of < 0.2 in multivariable analysis for PSA and ALP may indicate that there is true independent value in both factors, but the power of the current multivariable analysis was insufficient to prove it, due to the small sample size. Consequently, our results are only hypothesis-generating and need to be confirmed in further, larger studies.

## Conclusion

In this study, a score for easy clinical use to predict the treatment response to 2 cycles of RLT with ^177^Lu PSMA based on pretreatment PSA, ALP, and SUVmax of the “hottest lesion” in ^68^Ga PSMA-PET is presented, so that response rates of RLT can be increased and the rate of unnecessary adverse events can be reduced. This is a hypothesis-generating study, and further trials in independent, larger cohorts will be needed to test and validate this predictive score.
